# Rapid speciation in a newly opened postglacial marine environment, the Baltic Sea

**DOI:** 10.1186/1471-2148-9-70

**Published:** 2009-03-31

**Authors:** Ricardo T Pereyra, Lena Bergström, Lena Kautsky, Kerstin Johannesson

**Affiliations:** 1 Department of Marine Ecology -Tjärnö, University of Gothenburg, SE 452 96 Strömstad, Sweden; 2 Department of Botany, Stockholm University, SE 106 91, Stockholm, Sweden; 3 Institute of Coastal Research, Swedish Board of Fisheries, SE 742 22, Öregrund, Sweden

## Abstract

**Background:**

Theory predicts that speciation can be quite rapid. Previous examples comprise a wide range of organisms such as sockeye salmon, polyploid hybrid plants, fruit flies and cichlid fishes. However, few studies have shown natural examples of rapid evolution giving rise to new species in marine environments.

**Results:**

Using microsatellite markers, we show the evolution of a new species of brown macroalga (*Fucus radicans*) in the Baltic Sea in the last 400 years, well after the formation of this brackish water body ~8–10 thousand years ago. Sympatric individuals of *F. radicans *and *F. vesiculosus *(bladder wrack) show significant reproductive isolation. *Fucus radicans*, which is endemic to the Baltic, is most closely related to Baltic Sea *F. vesiculosus *among north Atlantic populations, supporting the hypothesis of a recent divergence. *Fucus radicans *exhibits considerable clonal reproduction, probably induced by the extreme conditions of the Baltic. This reproductive mode is likely to have facilitated the rapid foundation of the new taxon.

**Conclusion:**

This study represents an unparalleled example of rapid speciation in a species-poor open marine ecosystem and highlights the importance of increasing our understanding on the role of these habitats in species formation. This observation also challenges presumptions that rapid speciation takes place only in hybrid plants or in relatively confined geographical places such as postglacial or crater lakes, oceanic islands or rivers.

## Background

Speciation is one of the most fundamental processes in evolutionary biology. It is a process in which the within population variation transforms into distinguishable groups of individuals through the evolution of intrinsic reproductive barriers [1]. The speed at which this process happens is still intriguing and controversial [2] but estimates of speciation rates generally show that 10^5^–10^7 ^years (yrs) are needed for new species to evolve [3]. However, theory predicts that speciation can happen more quickly -often called "contemporary" or "rapid evolution"-, particularly in new or extreme environmental conditions where selection for adaptation is strong [2,4,5]. Despite these theoretical expectations, the evidence of rapid speciation is primarily limited to classical evolutionary models such as cichlid fishes in discrete geographical spaces as a Nicaraguan crater lake [6] where competition is expected to be high and reproductive isolation is likely to occur within, or Hawaiian fauna [7] where the high number of species are more likely to drive speciation [8]. Evidence is also provided from fruit flies under laboratory conditions [9], from homoploid and polyploid hybrid plants [10,11] and from anadromous sockeye salmon in which some degree of reproductive isolation evolved after 13 generations [12]. For marine species, support for rapid speciation derives from the "white" sticklebacks and although the evidence is consistent with a rapid species origin, the estimates of divergence time do not correspond with the glacial history of these systems [13]. Hence, the rapid foundation of new species in the marine environment remains to be proven.

In the marine realm, genetic divergence between populations is expected to evolve relatively slowly as recruits and propagules are readily transported by ocean currents [14]. Hitherto, time estimates for marine speciation events that agree with geological events confirm the expectations of slow speciation; for example, the reproductive isolation between sister lineages of marine shrimps was completed >3.5 million years ago (Mya) [15], after the rise of the Isthmus of Panama.

**Figure 1:**
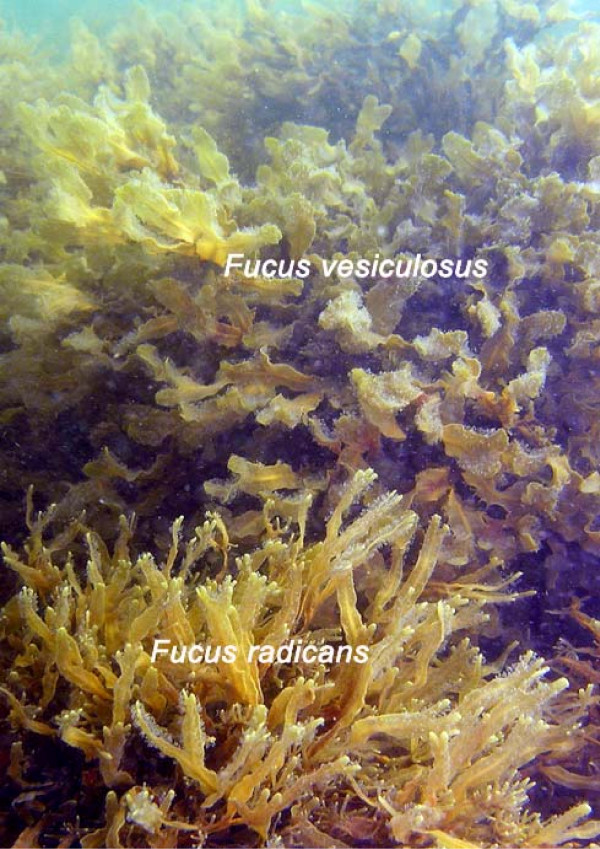
***Fucus radicans *and *F. vesiculosus***. Picture showing both *Fucus *species living in sympatry without any environmental discontinuity in the SW Gulf of Bothnia (northern Baltic Sea).

The Baltic Sea, today a large postglacial brackish-water basin of the NE Atlantic, hosts a low number of marine species. These species (together with a number of other now extinct ones), invaded the Baltic from the Atlantic during an earlier period of more marine conditions (4–8 thousand years ago, kya) and survived the shift 4 kya to the present day, where the low-saline environment (<10 practical salinity units, psu) creates an unusual marine ecosystem. One of these species, the bladder wrack *Fucus vesiculosus*, is widely distributed in the sub-Arctic and temperate regions of northern Atlantic, and is currently the most dominant and ecologically important perennial large brown alga in the Baltic [16]. In the Gulf of Bothnia (northern Baltic Sea), *F. vesiculosus *coexists with a smaller and morphologically distinct taxon, *Fucus radicans *[17]. *Fucus radicans *is smaller and has a bushy appearance in comparison to *F. vesiculosus*, and the thalli of *F. radicans *are always less wide than those of *F. vesiculosus *(Fig. 1). In contrast to the pan-Atlantic distribution of *F. vesiculosus*, *F. radicans *is endemic to the Baltic Sea. Previous analyses suggested that both taxa are reproductively isolated, but this conclusion was based on a single sympatric locality from the south part of the Gulf of Bothnia [17,18]. Subsequent studies could not, however, resolve the evolutionary relationships between *F. radicans *and *F. vesiculosus *using chloroplast (RuBisCO gene) [17] or mitochondrial (intergenic spacer) [19] DNA sequence markers. Therefore, the question is whether these two species correspond to previously diverged lineages that remained different after the Last Glacial Maximum 8 kya or if *F. radicans *evolved following the last ice age, presumably inside the Baltic Sea. Here, we supplemented previous studies with four additional sympatric sites inside the Baltic and with two sites outside this region as outgroups. We also increased the set of microsatellite loci to nine. Altogether, these provided us with tools to: **a) **assess the reproductive isolation between *F. radicans *and *F. vesiculosus *in the supplementary populations and with the additional loci; **b) **determine the phylogeographic affinities of *F. radicans*; **c) **estimate the time of divergence of *F. radicans *from *F. vesiculosus *to determine whether *F. radicans *originated recently, after the formation of the Baltic Sea and; **d) **to discern the mechanisms responsible for the reproductive isolation between *F. radicans *and *F. vesiculosus*.

## Results and discussion

### Reproductive isolation

**Figure 2:**
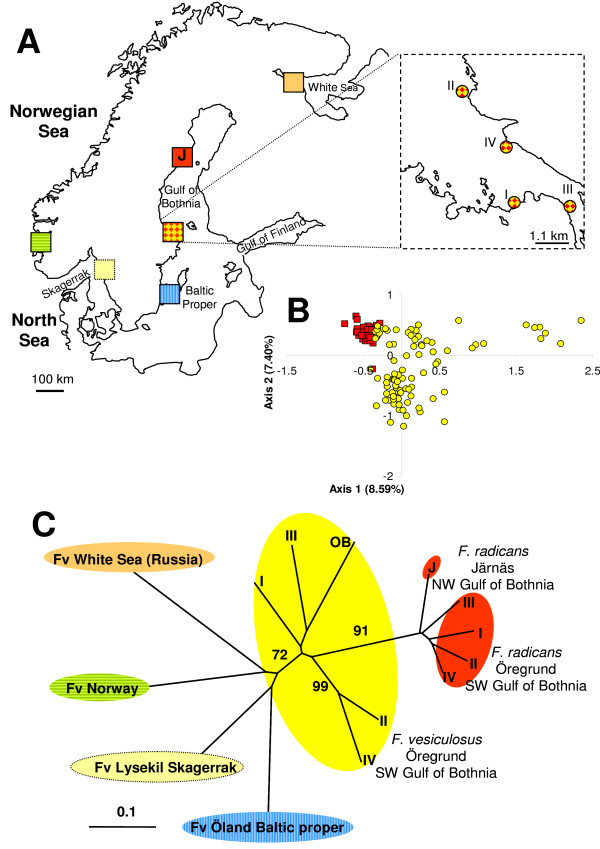
**Sampling localities and genetic differentiation of *Fucus *species**. A. Map of Scandinavia showing the main sampling localities and the sampling sites in Öregrund (SW Gulf of Bothnia), where both species occur in sympatry. B. Two-dimensional representation of a factorial correspondence analysis based on microsatellite genotype data. Genotypes are coloured according to species: *F. radicans *(red squares) and *F. vesiculosus *(yellow circles). C. Neighbour-joining microsatellite-based population tree calculated with Cavalli-Sforza genetic distances. Fv denotes *F. vesiculosus*.

**Figure 3:**
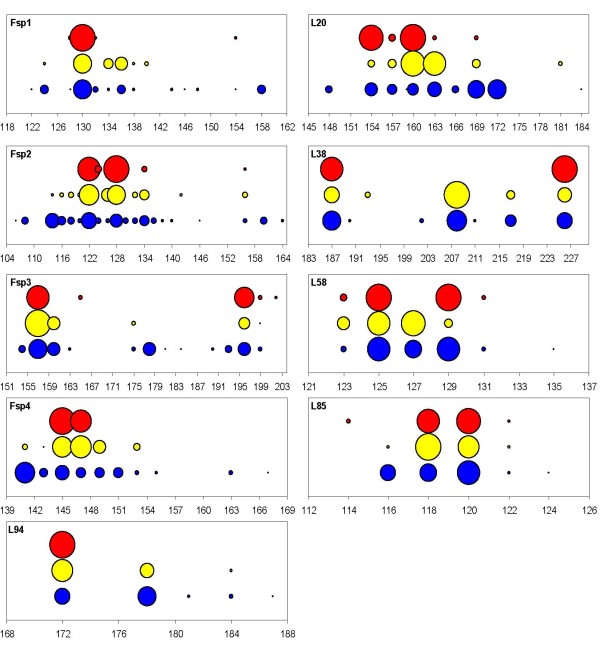
**Allele frequency distribution of microsatellite loci**. Each circle represents an allele and its frequency is proportional to the size of the corresponding circle (red: *F. radicans*, yellow: *F. vesiculosus *in Öregrund, SW Gulf of Bothnia; blue: *F. vesiculosus *in all other populations).

**Figure 4:**
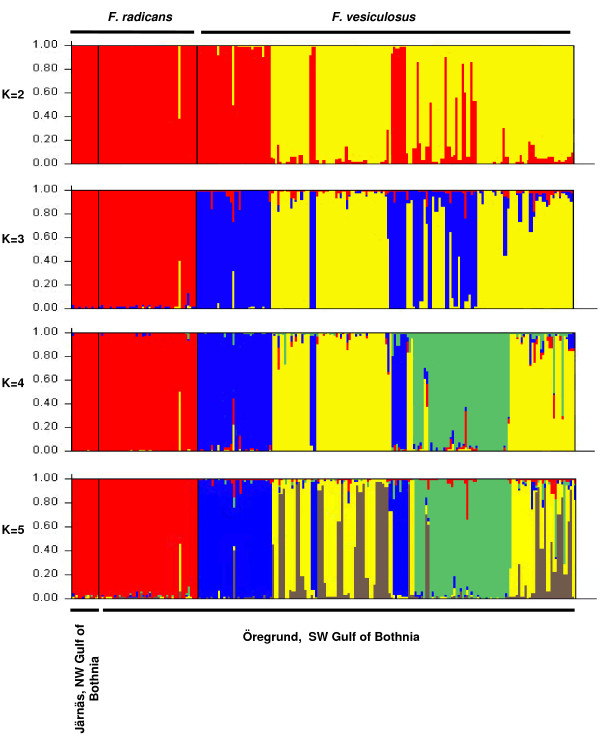
**Histogram of Bayesian assignment tests**. Analysis based on genotypes from individuals of *F. radicans *and *F. vesiculosus *exclusively from the Öregrund (SW Gulf of Bothnia) sampling sites, where both species occur in sympatry. Each bar represents an individual and its assignment probability into one of *K *clusters. Samples are without repeated genotypes assumed to be clones. More than one color per individual indicates admixture.

**Figure 5:**
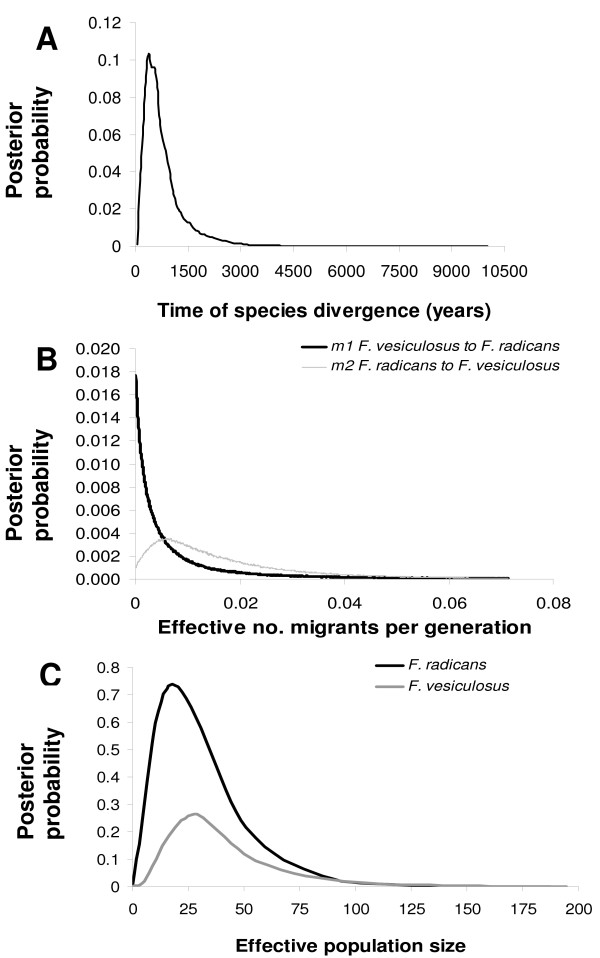
**Approximate Bayesian posterior probability distributions for demographic parameters**. A. Estimate of time since divergence (*t*) in years. B. The number of effective migrants per generation from *F. vesiculosus *to *F. radicans *and vice versa. C. Estimate of effective population sizes for both species.

We assessed the reproductive isolation in four sympatric populations of *F. radicans *and *F. vesiculosus*, where both taxa are frequent in SW Gulf of Bothnia (Fig. 2A) and with an increased set of microsatellite loci (9 instead of 5 from the earlier study [17]). Only individuals firmly attached to the bottom and with attached representatives of the other taxon within a distance of <1 m were used in the analyses. However, the individuals representing identical genotypes (clones) were removed for all subsequent analyses (see Methods). Thus, in total we used 235 *Fucus *individuals with unique genotypes. All microsatellite loci were polymorphic showing 5–21 different alleles per locus and heterozygosity values (*H*_e_) ranging from 0.101 to 0.827 in both *Fucus *species (table 1). As for the reproductive isolation of *F. radicans*, considerable differences are evident from the allele frequency distributions across loci, particularly at loci Fsp1, L38, L58, and L94 (Fig. 3). At least one allele represented with high frequency in each locus in *F. vesiculosus *is not present in *F. radicans*, showing marked differences between species. Subsequently, we performed a factorial correspondence analysis to assess the species cohesion without accounting for any historic or demographic pattern that may underlie the taxa identities. These results showed a clear assembly among *F. radicans *individuals, while some *F. vesiculosus *individuals appeared scattered and a few more overlapping the *F. radicans *grouping (Fig. 2B). These overlapping individuals suggest that the species divergence might have occurred in the presence of gene flow, but without any demographic information this suggestion should be interpreted with caution. However, to determine whether this overlapping is due to morphological misclassification or the presence of hybrids we inferred individual ancestry using a Bayesian assignment analysis. Results from this analysis showed a clear separation between *F. radicans *and *F. vesiculosus *individuals with additional genetic variation in the latter that includes the potentially misclassified individuals. This clustering, however, had no discernable geographic pattern in *F. vesiculosus *but it separated unambiguously those *F. vesiculosus *individuals that seem to overlap with *F. radicans *from all the *F. radicans *individuals (Fig. 4, *K *= 3). It is also important to highlight the genetic cohesiveness amongst *F. radicans *across 550 km of coastline from Järnäs to Öregrund, providing further support of genetic isolation from *F. vesiculosus*. To further determine the extent of gene flow within and between species populations we used *F*_ST _estimates, which are commonly used to investigate the magnitude of population differentiation. From this analysis, it is clear that the sympatric populations of *F. radicans *and *F. vesiculosus *identified by morphological criteria (frond width, midrib width and stipe width) [17] were indeed genetically isolated in the SW Gulf of Bothnia (table 2). An overall *F*_ST _= 0.160 (*P *= 0.05) among species' populations indicates limited gene flow between *F. radicans *and *F. vesiculosus*. Furthermore, all *F. radicans *populations were significantly different from those of *F. vesiculosus *and low, non-significant values characterize *F. radicans *populations, showing added evidence of reproductive isolation. Large genetic variation was also evident from these estimates among *F. vesiculosus *populations in agreement with previous results showing constrained gene flow in this species over short distances [20]. Yet, the differences between *F. radicans *and *F. vesiculosus *at all sympatric localities were greater than those observed within *F. vesiculosus*. Further results of the extent of gene flow between species comes from coalescent – based pairwise estimates of migration between both species' populations and effective population sizes. The 95% highest probability distributions (HPD) of the number of migrants per generation showed asymmetric gene flow (Fig 5B–C). Migration values from *F. vesiculosus *to *F. radicans *had their highest probability near zero suggesting the absence of gene flow in this direction (*m*1 = 1.01 × 10^-4 ^– 2.5 × 10^-2^). In contrast, low but non-zero gene flow was detected from *F. radicans *to *F. vesiculosus *(*m*2 = 1.16 × 10^-3 ^– 2.7 × 10^-2^), supporting the suggestion of recent divergence.

**Table 1:** Genetic variability among microsatellite loci examined

	***F. radicans*** **(n = 39)**	***F. vesiculosus*** **(n = 117)**	***F. vesiculosus other populations*** **(n = 123)**	***Total***
**Fsp1**				
NA	4	6	14	**14**
*H*_O_	0.051	0.550*	0.537	
*H*_e_	0.101	0.636	0.740	
**Fsp2**				
NA	5	12	21	**21**
*H*_O_	0.846*	0.362*	0.306*	
*H*_e_	0.571	0.780	0.827	
**Fsp3**				
NA	5	7	13	**14**
*H*_O_	0.615	0.388*	0.499*	
*H*_e_	0.587	0.522	0.816	
**Fsp4**				
NA	2	6	10	**10**
*H*_O_	0.538	0.713	0.659	
*H*_e_	0.582	0.708	0.729	
**L20**				
NA	5	7	11	**11**
*H*_O_	0.615	0.225*	0.574	
*H*_e_	0.567	0.655	0.774	
**L38**				
NA	2	6	8	**8**
*H*_O_	0.641	0.338*	0.513	
*H*_e_	0.501	0.651	0.648	
**L58**				
NA	4	5	6	**6**
*H*_O_	0.897*	0.525	0.597	
*H*_e_	0.555	0.648	0.620	
**L85**				
NA	4	4	6	**6**
*H*_O_	0.718	0.575	0.501	
*H*_e_	0.567	0.593	0.607	
**L94**				
NA	1	3	5	**5**
*H*_O_	-	0.275*	0.336	
*H*_e_	-	0.454	0.536	

Sample sizes in "*F. radicans*" and "*F. vesiculosus*" only correspond to sympatric samples from Öregrund and using exclusively unique genotypes. Number of alleles (NA), observed (*H*_o_) and expected (*H*_e_) heterozygosities at each microsatellite locus. Significant deviations from Hardy-Weinberg equilibrium after Bonferroni correction are indicated by asterisks.

**Table 2:** Pairwise F_ST _values of population differentiation

	*Fr *(J)	*Fr *(I)	*Fr *(II)	*Fr *(III)	***Fv *(I)**	***Fv *(II)**	***Fv *(III)**	***Fv *(IV)**
*Fr *(J)	-							
*Fr *(I)	0.0189	-						
*Fr *(II)	0.0013	0.0164	-					
*Fr *(III)	0.0019	0.0454	0.0088	-				
***Fv *(I)**	0.2305*	0.1864*	0.2043*	**0.1889**	-			
***Fv *(II)**	0.1980*	0.1854*	0.1741*	0.1541*	0.0946	-		
***Fv *(III)**	0.2197*	0.1901*	0.1848*	**0.1780**	0.1539*	0.1694*	-	
***Fv *(IV)**	0.2839*	0.2650*	0.2543*	**0.2348**	0.1554	0.1004	0.1650	-

*F*-statistics were calculated among collecting sites in Öregrund (SW Gulf of Bothnia) where both *Fucus *species occur in sympatry. Asterisks indicate significant *P*-values after Bonferroni correction (*P *≤ 0.05). Abbreviations indicate *F. radicans *(*Fr*) and *F. vesiculosus *(*Fv*). Roman numbers indicate the collecting sites according to Fig. 2A.

### Phylogeographic affinities of *F. radicans*

We constructed a neighbour-joining tree based on Cavalli-Sforza genetic distances adding samples from one allopatric locality of *F. radicans *and one of *F. vesiculosus *from inside the Baltic, and three localities of *F. vesiculosus *from outside the Baltic (North Sea, Norwegian Sea and White Sea) (Fig. 2A). This analysis showed *F. radicans *emerging as a single monophyletic taxon derived from a *F. vesiculosus *lineage, but distinct from *F. vesiculosus *with high bootstrap support (Fig. 2C). The population tree indicated a close relationship between *F. radicans *and Gulf of Bothnia *F. vesiculosus*, suggesting that *F. radicans *recently diverged from this *F. vesiculosus *lineage. Further clustering of *F. vesiculosus *populations mainly corresponds to geographical designations and the genetic distances between them are in agreement with a previous study suggesting that this divergence reflects constrained gene flow even at small geographic scale [20].

### Time of divergence

An alternative scenario to the hypothesis of recent speciation is that *F. radicans *may have originated outside the Baltic and entered the newly formed sea as a previously diverged lineage that remained differentiated (and became extinct outside the Baltic Sea). This scenario is weakened by our phylogeographic data: our neighbour-joining tree showed a close relationship of *F. radicans *with Baltic populations of *F. vesiculosus *that strongly supports an *F. radicans *origin within the Baltic. Yet, accurate times of speciation are difficult to estimate due to the lack of variation in sequence loci. Neither nuclear or mtDNA sequences were able to resolve the phylogenetic relationships between both taxa [17,19,21], further suggesting a recent origin of *F. radicans*. Consequently, we calculated a microsatellite-based estimate of time since divergence of *F. radicans *and *F. vesiculosus *using a coalescent approach. This analysis indicated that *F. radicans *and *F. vesiculosus *started to diverge from a common panmictic population sometime between 125 and 2475 yrs ago (95%HPD; posterior distribution peak at ~400 yrs ago, Fig. 5A). Hence, separation took place after the Baltic underwent the transition from marine to brackish water, less than 4 kya. The hypothesis that *F. radicans *arouse recently is further strengthened by the fact that it is endemic to the Baltic Sea.

### Isolating mechanisms

We considered the mating system as a potential isolating mechanism between *F. radicans *and *F. vesiculosus*. *Fucus vesiculosus *has separate sexes and was until recently reported to reproduce exclusively sexually through external fertilization. Experiments show limited capacity of *F. vesiculosus *to reproduce in low salinities by reducing the longevity and motility of the gametes [22,23], low fertilization success and egg polyspermy [22]. However, asexual reproduction (20%) has also been reported in Baltic populations of *F. vesiculosus *[18]. Likewise, *F. radicans *has permanent and well established populations in all its distributional range, with separate sexes and sexual reproduction taking place the same way as in *F. vesiculosus*. However, it shows high extent of clonality and re-attachment experiments in both species show that detached thallus fragments of *F. radicans *have considerably higher capacity to re-attach (80%) than those of *F. vesiculosus *(15%) [18].

Populations living in marginal environments typically switch or are capable of asexual reproduction [24], and this is also true for several species in the Baltic [25]. Thus, the frequent clonal reproduction observed in *F. radicans *coupled with the low capacity of *F. vesiculosus *to reproduce sexual or asexually at low salinities may have facilitated the divergence between both taxa. The evolution of low-salinity tolerance might be seen as directional selection in *F. radicans *and *F. vesiculosus*. Clonality may have evolved through a single *F. radicans *individual successfully colonizing and producing a population in the hypo-saline environment or through reinforcement to reduce gene flow from *F. vesiculosus *populations not adapted to these conditions. In either case, reproductive isolation would appear as a by-product of adaptation.

The Baltic is an ecologically marginal and geographically peripheral marine habitat due to its permanent low salinity and geographic semi-isolation from the Atlantic. The salinity gradient from the inner Baltic to the North Sea spans an order of magnitude (3–30 psu), and has caused strong local adaptation in most of the marine lineages that survived the marine/brackish transition 4 kya [25]. Directional selection is a strong promoter of speciation, even in the presence of gene flow [26-28]. More specifically, environmental stress along gradients has been highlighted as a potential source of new species [29]. Although the exact mechanism of the *F. vesiculosus *– *F. radicans *speciation event remains unknown, the extreme environmental stress imposed by the brackish water environment of the Baltic has clearly contributed to the formation of the new species.

## Conclusion

*Fucus radicans *is endemic to the Baltic Sea that formed only 8–10 kya. This species diverged from *F. vesiculosus *and divergence time estimates suggest that they split about 400 yrs ago. These dates are consistent with the transition of the Baltic from marine to brackish water, less than 4 kya and provide an unparalleled example of rapid speciation in marine ecosystems. These closely related species also offer further opportunities to increase our understanding of the role of species-poor systems -where competition is low and gene flow is expected to be high-, of peripheral extreme environments and of mixed reproductive modes in species formation.

## Methods

### Sample collection

Individuals of *F. radicans *and *F. vesiculosus *were collected from four different areas along the Swedish coast. The area of Öregrund (SW Gulf of Bothnia) included four sites in which the distributional ranges of both species overlap and individual plants occur in sympatry (Figs. 1, 2A and 2C). The following additional sites were also included: one sampling site at Järnäs (NW Gulf of Bothnia) where only *F. radicans *is found; Öregrund (OB, *F. vesiculosus*, n = 37, Fig. 2C); Öland (n = 43, Baltic) and Lysekil (n = 42, Swedish west coast) where only *F. vesiculosus *occurs. Two further populations of *F. vesiculosus *were sampled from Norway (n = 20) and the White Sea (n = 18) for use as outgroups. Total number of unique genotypes used for the analyses is provided in table 1.

### Genotyping

DNA was extracted from dried algal tissue using DNeasy Plant MiniKit and samples were genotyped at nine microsatellites developed from *Fucus *species [30,31]. Labelled products were poolplexed and resolved on a Beckman-Coulter automated sequencer and CeqMan 8000 software (Beckman-Coulter) was used for allele sizing.

### Summary statistics

For each species, the probability of identity of genotypes was calculated to distinguish between clones and identical genotypes by chance using GIMLET . The individuals representing clones were removed for all subsequent analyses. Allele variation and genetic diversity were obtained with POP100GENE  (table 1 and Fig. 3). Tests for linkage disequilibrium, Hardy-Weinberg departures and their statistical significance were performed using GENEPOP 4.0 .

### Population differentiation and Bayesian population assignment test

First, to identify and illustrate in the factorial space the degree of similarity in allelic states between populations of both taxa from Öregrund, where they occur in sympatry, a factorial correspondence analysis (FCA) was carried out using GENETIX 4.03  (Fig. 2B). Subsequently, to examine whether sympatric populations of *F. radicans *and *F. vesiculosus *are genetically different and to measure the difference magnitudes, *F*-statistics were calculated using FSTAT 2.9.3  and significance levels were Bonferroni-corrected (table 2). Then, to assess the genetic affinities between species and populations a neighbour-joining tree was constructed using Cavalli-Sforza genetic distances with 10,000 bootstrap support replicates on locus information using POPULATIONS  (Fig. 2C). Finally, to provide an alternative classification of individuals and identification of potential hybrids, a Bayesian assignment analysis was also performed using STRUCTURE 2.2  with a burn-in period of 50,000 and 1,000,000 iterations. The algorithm infers individual ancestry by assigning sampled individuals into a user-defined number of clusters (K)/populations that minimize genotypic disequilibrium under the assumption of random mating. The maximum number of clusters was set to *K *= 5, (Fig. 4).

### Estimation of demographic parameters of species divergence

**Figure 6:**
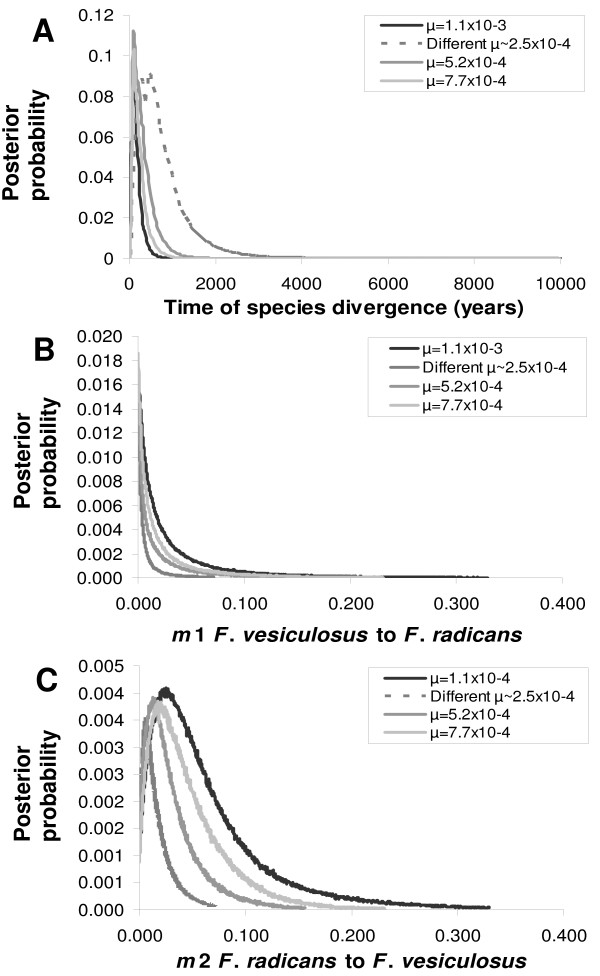
**Approximate Bayesian posterior probability distributions for demographic parameters using different microsatellite mutation rates**. A. Estimate of time since divergence (*t*) in years. B. The number of effective migrants per generation from *F. vesiculosus *to *F. radicans *(*m*1). C. The number of effective migrants per generation from *F. radicans *to *F. vesiculosus *(*m*2).

Coalescent-based estimates of time since divergence of the two species and effective number of migrants per generation were calculated using IM . Asymmetrical gene flow (*m*1 ≠ *m*2) was allowed because preliminary runs indicated that migration rates were different. Wide uninformative prior distributions were assigned based on three preliminary trial runs. Metropolis coupling was implemented using 20 chains with a 20 chain swap attempts per step and a geometric heating increment. A burn-in period of 500,000 steps was used and results were recorded every hour for >20,000,000 steps, so that the lowest effective sample sizes (ESS) for each parameter were 500 [32]. To test for the performance of the program, the analysis was conducted five times under identical parameterization but with different random number seeds. Given that the mutation is critical to convert the coalescent estimates to biologically informative demographic parameters, a series of analyses were conducted using different mutation rates to establish a confidence interval of coalescent estimates (Fig. 6). The upper (5.2 × 10^-4^) and lower (1.1 × 10^-3^) 95% confidence limits of mutation rate for nuclear microsatellite loci of plants [33] were used as reference. A point estimate of 7.7 × 10^-4 ^for microsatellite with dinucleotide repeats was also used from plants. However, since our data comprises microsatellite loci with di- and trinucleotide repeats, mutation rates for each locus used in our study were calculated indirectly using Msvar 1.3 . These mutation rates (2.5 × 10^-4 ^geometric mean) produced the most conservative coalescent estimates from IM and therefore are the ones presented in the main results (Fig. 5). Finally, a generation time of 6 yrs [34] was assumed to convert the coalescent estimates to demographic parameters.

## Authors' contributions

RTP, LB, LK and KJ designed research. LB and LK conducted field collection, RTP performed molecular and statistical analysis and drafted manuscript, KJ participated in drafting the manuscript. LB and LK revised critically the manuscript. All authors read and approved the final manuscript.
